# Hernia Sac Preservation for Prevention of Transversus Abdominis Release in Laparoscopic Extended-Totally Extra Peritoneal Repair of Ventral Hernia: A Minimalistic Solution for a Formidable Challenge

**DOI:** 10.3389/jaws.2022.10634

**Published:** 2022-11-17

**Authors:** Premkumar Balachandran, Subbiah Tirunelveli Sivagnanam, V. C. Swathika

**Affiliations:** ^1^ Institutes of Hernia Surgery and Abdominal Wall Reconstruction, Apollo Hospitals, Chennai, India; ^2^ Department of General Surgery, Apollo Speciality Hospital, Chennai, India

**Keywords:** transversus abdominis release, laparoscopic ventral hernia repair, E-TEP, hernial sac preservation, abdominal wall reconstruction

## Abstract

**Background:** Ventral hernia repair has always been an extensive and challenging surgery. The laparoscopic extended—Totally Extraperitoneal (E-TEP) technique of ventral hernia repair is gaining popularity due to the advantage of placing a large mesh in the retro rectus plane. When done through a Laparoscopic approach, the difficulty of the procedure is compounded by multiple factors such as obtaining retro muscular access, maintaining the retro muscular plane, crossing over to the contralateral retro muscular plane without entering intraperitoneally, suturing in a limited space, and manipulation of a large mesh in a constricted space for placement. In cases of large midline incisional hernias, dense adhesions to the previous surgical scar are often present. Despite having extremely satisfying outcomes, the aforementioned factors make the laparoscopic extended—total extraperitoneal repair of large midline ventral and incisional hernias an exceptionally challenging procedure. A tension-free midline approximation is the benchmark of ventral/incisional hernia surgery. In certain cases, this can be difficult to achieve due to multiple factors. For the purpose of attaining tension-free midline closure, component separation techniques (CST) have been explored and implemented. Of these, the posterior component separation technique of Transversus Abdominis Release (TAR) has gained popularity for reducing the tension of posterior rectus sheath during posterior midline closure in retro muscular repairs by adding a few centimetres of medial advancement. The main pitfall of TAR is its technical complexity, which may result in morbid complications when implemented incorrectly. Performing TAR laparoscopically compounds the complexity manyfold. Hence, to obviate the necessity to perform Laparoscopic TAR in cases of Laparoscopic E-TEP repair of large midline ventral and incisional hernias, we present that the technique of hernial sac preservation should be pre-emptively carried for all Laparoscopic ETEP repairs so that the necessity of performing TAR in select cases is reduced by aiding in the addition of final crucial centimetres of lengthening to the posterior rectus sheath for achieving posterior midline closure. This aids in the success of the procedure by preventing an additional complex procedure of TAR from being performed in an already challenging hernia repair technique of Laparoscopic E-TEP repair.

**Methods:** We hereby report three cases of Ventral hernia repair in which Laparoscopic E-TEP repair was carried out and Hernial sac preservation technique was implemented successfully. Midline closure of the posterior rectus sheath was attained under reduced tension and a medium-weight macroporous polypropylene mesh was placed in the retro-rectus plane after measurement of the potential space. Patients were discharged uneventfully.

**Results:** Patients were followed up for up to 6 months postoperatively and were found to have no complications.

**Conclusion:** In Laparoscopic E-TEP repair of midline ventral hernias, preservation of the hernial sac along with the posterior rectus sheath might aid in the prevention of performing a TAR in selected cases where posterior layer tension is present. Hernia sac preservation thereby aids in reducing operative time and preventing potential morbid complications.

## Introduction

Evolving studies about abdominal wall reconstruction have proved that a repair using a mesh is the ideal repair of a hernia [1]. Placement of various meshes at various sites has been assessed and the retro-muscular plane has been proven to be a safe space for mesh placement [2].

With the minimal access approach to hernia repair becoming mainstream worldwide, the Extended—Totally extraperitoneal (E-TEP) repair of hernias has gained prominence [3]. The dynamics of the abdominal wall muscles have been better understood and placement of mesh in the retro-rectus plane has been demonstrated to be favourable, due to the mesh not being exposed to the subcutaneous fat or bowel as in overlay and intraperitoneal onlay mesh repairs, respectively.

Laparoscopic E-TEP repair entails technically precise and demanding manoeuvres, often requiring specialized training under an experienced surgeon-mentor, intracorporeal suturing and fine dissection skills. Access into the retro muscular space is attained by open technique or by using optical access trochars. In our experience, accessing the correct plane on initial entry has been an essential step in maintaining the correct plane throughout the procedure and allowing for easier dissection aided by insufflation. The limited space in the retro rectus plane adds to the complexity of crossing over to the contralateral retro rectus space for continuing the inferior dissection. In the case of large midline incisional hernias, the adherence of the tissue to the previous surgical scar adds to the difficulty in dissection and resuming it without entering the peritoneal cavity on damaging the posterior rectus sheath. Closure of rents in the posterior surface entails suturing in a limited space. Closure of the defect entails extensive suturing on the roof which by itself is a complex manoeuvre. Once the posterior layer continuity is attained, a large medium-weight macroporous polypropylene mesh is placed and unrolled inside the retro rectus cavity. The abovementioned steps in brief entail the complexity of laparoscopic E-TEP repair of midline ventral and incisional hernias.

Posterior rectus sheath closure under reduced tension is an essential step in laparoscopic E-TEP repair. In case of large defects, of EHS W2 [4] or more, attaining continuity of the posterior layer makes it difficult to achieve tension-free primary closure. For this purpose, a laparoscopic Transversus Abdominis Release [5] (TAR) is performed on one side or bilaterally depending on the defect size requiring closure. Just medial to the neurovascular bundles, the posterior lamella of the internal oblique is incised and the transversus abdominis muscle is visualized. A hook is used to divide the fibers of the transversus abdominis muscle and the procedure is continued distally/proximally. Inferior to the arcuate line, only the transversalis fascia and the peritoneum are medialized. Care must be taken not to damage the neuro-vascular bundles, whose injury will denervate the rectus muscle and lead to further deformity of the abdominal wall [6, 7].

The above technique of Laparoscopic transversus abdominis release, a complex procedure in its own right, adds to the already demanding and substantial procedure of laparoscopic E-TEP repair of midline ventral and incisional hernias.

We propose that the technique of hernia sac preservation, a technique already in use for retro muscular repairs, should be done routinely for all Laparoscopic E-TEP ventral and incisional hernias to add to the length of the posterior layer in continuity with the posterior rectus sheath and aid in the prevention of a TAR being performed in selected cases, thereby reducing the time and complexity of the laparoscopic ventral/incisional hernia repair.

## Methods

We hereby report three cases of Laparoscopic ventral hernia repair in which Extended—Totally Extraperitoneal repair (E-TEP) was performed with the implementation of hernial sac preservation technique.

**FIGURE 1:**
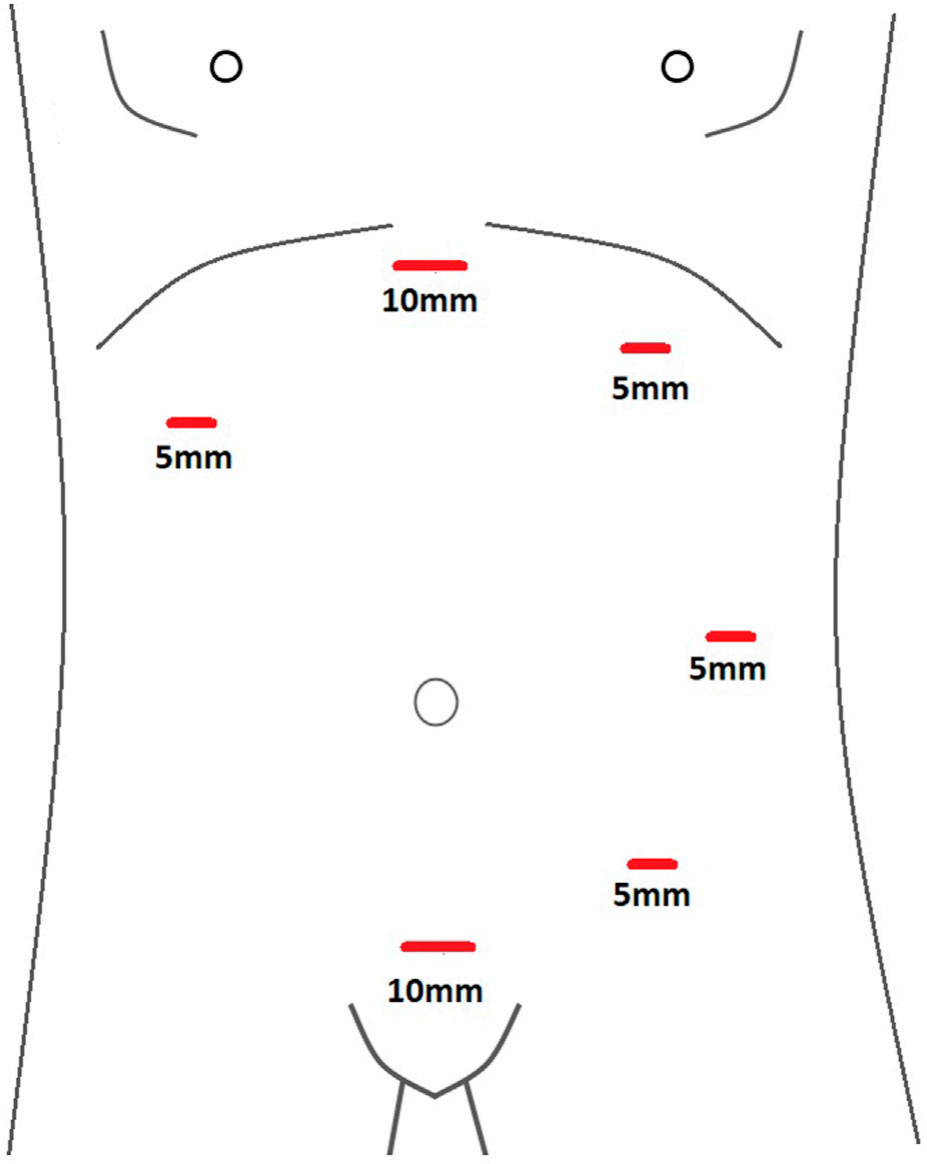
Schematic diagram of port placement used for Laparoscopic ETEP repair of Midline incisional and ventral hernias.

**FIGURE 2:**
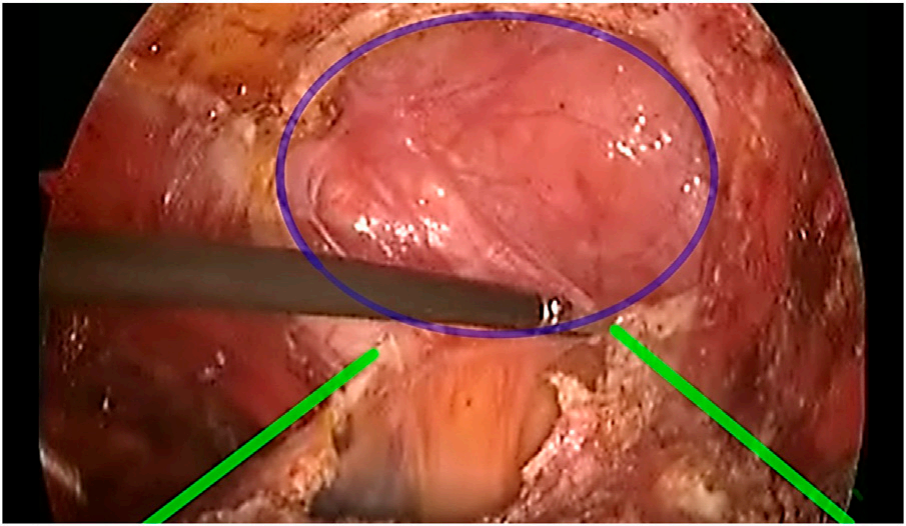
Defect (Blue) demonstrated with the edges of the Posterior rectus sheath (Yellow) being dissected out.

**FIGURE 3:**
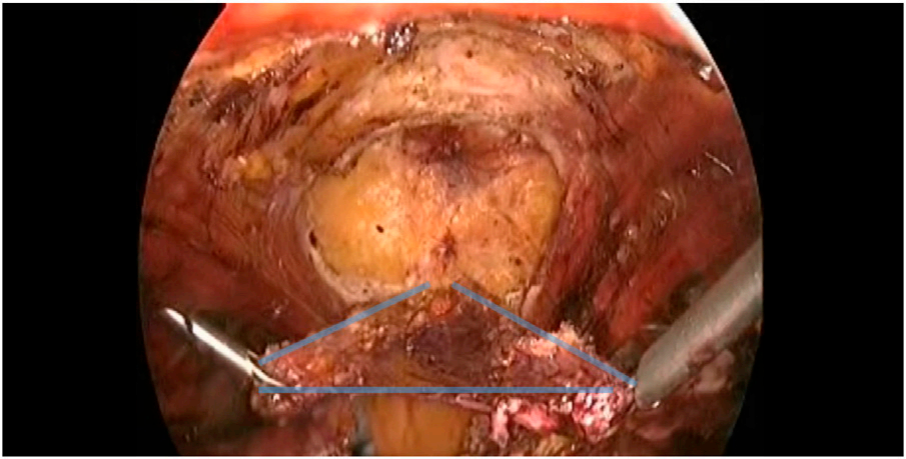
Demonstrating the preserved sac (Blue border).

**FIGURE 4:**
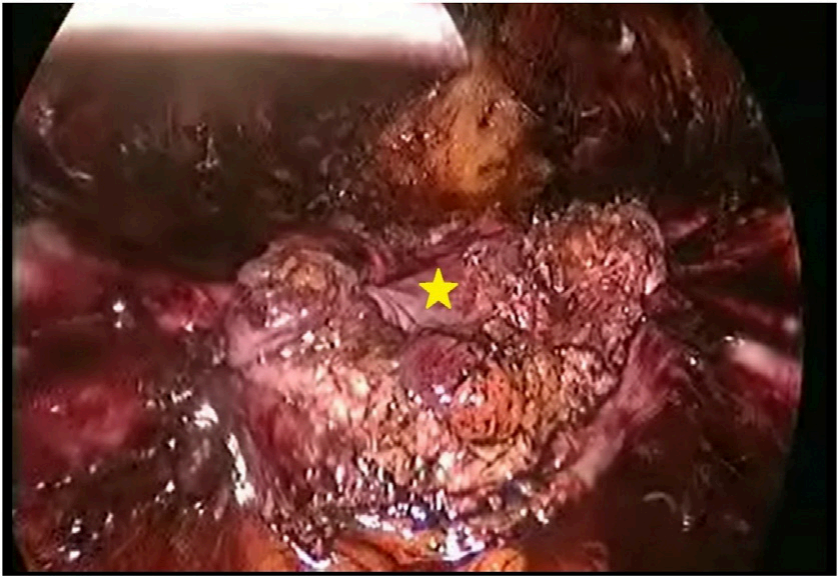
Hernial sac forming a continual layer along with the peritoneum aiding in midline closure under reduced tension (*).

**FIGURE 5:**
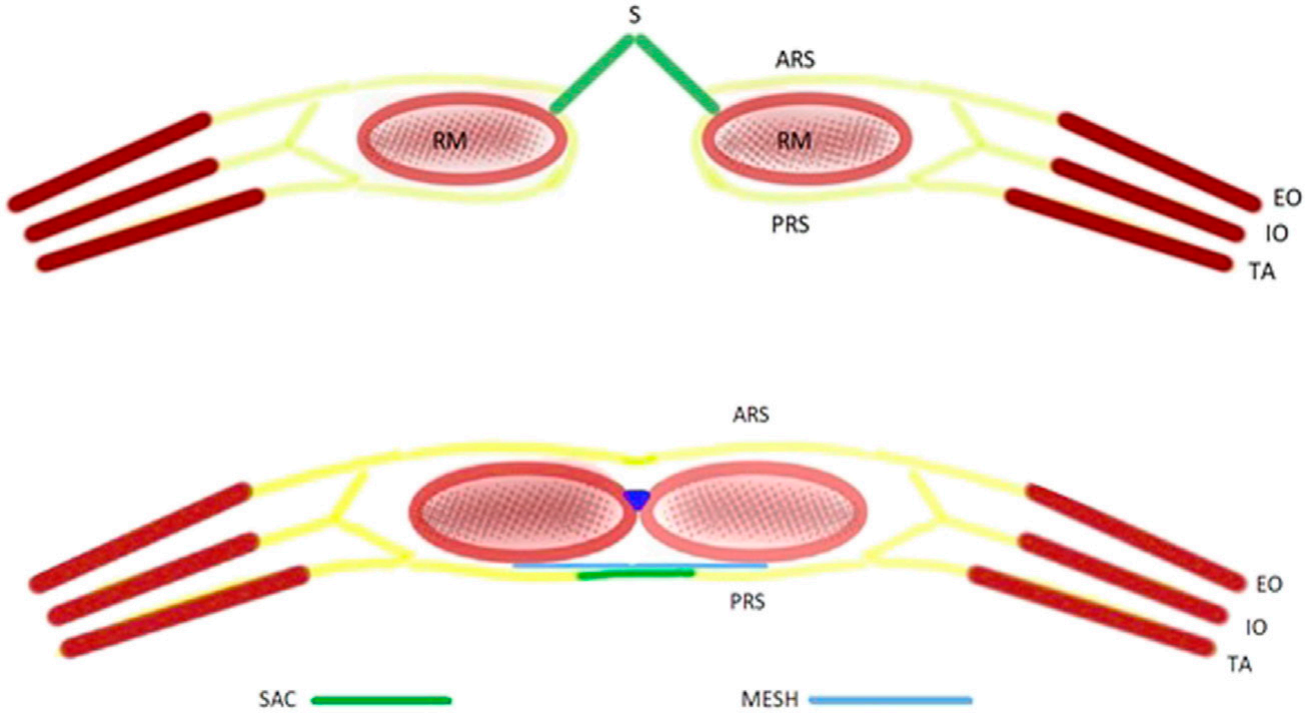
Schematic diagram of the Hernial sac preservation technique. (RM, rectus muscle; EO, external oblique; IO, internal oblique; TA, transversus abdominis; ARS, anterior rectus sheath; PRS, posterior rectus sheath).

A 75-year-old female (Patient 1) with an EHS M4 W2 hernia was planned for E-TEP ventral hernia repair. She had undergone a Total abdominal Hysterectomy with bilateral salpingo-oophorectomy 14 years back by an infraumbilical vertical midline incision and developed swelling over the previous surgical site 5 years back, which has been increasing in size ever since. A contrast-enhanced computed tomography was performed and revealed a defect width of 7.2 cm. A presurgical workup revealed no existing co-morbid illnesses. The retro rectus plane was entered using optical trochar in the left upper quadrant, medial to the left midclavicular line and was insufflated with CO_2_ (Figure 1). Ipsilateral telescopic blunt dissection of the left retro-rectus space was carried out until the pubic bone was visualized. Two lateral working ports medial to the linea semilunaris were inserted in the left lumbar and left iliac fossa under vision. Lateral and inferior dissection was carried out using a monopolar spatula and the neuro-vascular bundles were visualized and preserved. Superior medial cross-over was done and the contralateral rectus muscle was visualized. A 10 mm port was inserted in the epigastric region and the endoscope was shifted towards it to provide a top-down view for inferior dissection. A 5 mm working port was inserted in the right upper quadrant to aid in ergonomic dissection caudally. Dissection was carried out till the neuro-vascular bundles on the contralateral side were identified. Inferior dissection was carried out towards the pubic bone. The contents of the hernia were reduced and the sac was dissected out from the defect in continuity with the posterior rectus sheath. A suprapubic 10 mm port and a 5 mm port in the right iliac fossa were made to facilitate dissection in the epigastric region. The proximal cut edges of the posterior rectus sheath which were separated from the midline could be brought together only under tension (Figure 2). Hence the hernial sac preservation was attempted and implemented (Figures 3, 4, 5). The defect on the anterior abdominal wall was closed with 0′–30 cm V-Loc™ barbed sutures [delayed absorbable copolymer made of glycolic acid and trimethylene carbonate [8]]. The preserved hernia sac was used to approximate the posterior layer using 2-0′ V-Loc™ barbed sutures. The posterior rectus sheath along with the preserved hernial sac ensured a posterior layer under reduced tension. Rectus diastasis was also closed with 0′- V-Loc™ barbed sutures. The potential space was measured and a 30 cm × 15 cm medium weight macroporous polypropylene mesh was introduced through the suprapubic 10 mm port, unrolled and placed. No mesh fixation was done. 20F drain was placed in the retro rectus plane. The cavity was desufflated under vision ensuring that there was no folding or crumpling of the mesh. Ports were removed after ensuring hemostasis.

The above-mentioned technique was replicated in two other patients. A 66-year-old female (Patient 2) with an EHS M4 W2 hernia with a defect width of 4.8 cm, with a previous history of puerperal sterilization done through a suprapubic transverse incision 34 years ago and well-controlled Type 2 diabetes mellitus. The other patient was a 56-year-old female (Patient 3) with an EHS M3 W2 hernia with a defect width of 6.4 cm, a known hypertensive on medical treatment and no previous surgical history. In both of these patients, a tension-free midline closure of the posterior layer was achieved by preserving the hernial sac along with the posterior rectus sheath. Similarly, a 30 cm × 15 cm medium-weight macroporous polypropylene mesh was placed in the retro rectus plane and was not fixed. Defect closure was done using 0′–30 cm V-Loc™ barbed sutures. The technique of hernial sac preservation in Laparoscopic ETEP ventral hernia repair was replicated without TAR to achieve tension-free midline closure in the posterior layer.

The retro rectus drains were monitored and were removed on the 4th, 3rd and 4th postoperative days respectively of patients 1, 2 and 3. All three patients were discharged without any significant postoperative events.

## Results

All three patients recovered well postoperatively and were discharged on the 5th postoperative day after drain removal. They were advised to avoid weightlifting and to prevent coughing, constipation and straining during voiding. They were also advised to wear an abdominal corset for a period of 3 months postoperatively.

Patients were followed up for 6 months postoperatively and were found to have no complications.

## Discussion

With mesh repair of hernias, the hallmark of ideal hernia surgery is tension-free closure of the defect which is now accepted as the standard of care [9, 10]. As technical innovations in imaging, transmission and energy sources advance rapidly, the repair of hernias has now moved into an era of minimal access, where the advantages of clear and magnified vision, less postoperative wound infections, reduced postoperative pain, advances in instrumentation, reduction in length of hospital stay and earlier return to daily activities have made laparoscopic hernia surgery the treatment of choice across the world [11].

Sublay retro muscular repairs have gained prominence, such as the E/MILOS repair [12] and TARM [13] (Trans Abdominal Retro Muscular repair) due to the unique advantage of the placement of a regular polypropylene mesh and avoidance of usage of expensive dual layer meshes. In our centre, we utilize the technique of extended Total extraperitoneal repair for ventral hernias due to better expertise in performing this procedure.

Peritoneal flap hernioplasty [14] is another technique that can be used to achieve tension-free midline approximation. In comparison to the hernia sac preservation technique, it is more technically complex and time-consuming as it involves division of the sac in the midline and attachment of the cut ends to the anterior and posterior rectus sheath.

The advent of the Laparoscopic Extended-Totally Extraperitoneal approach for treatment of Ventral hernia has gained significant popularity. In E-TEP, a large retro-muscular space is available [3] which allows a larger mesh to be placed and allows for a wide mesh overlap over the entire hernial defect. Since the dissection in E-TEP is done inferiorly till the space of retzius, other missed ventral hernias or those with swiss-cheese type defects can also be repaired in the same procedure. This is a unique advantage of this technique and has presented great outcomes as the mesh is placed away from the bowel in the retro-rectus plane.

In the retro-rectus repair of large hernias after reduction of contents, it is often a formidable task to achieve tension-free closure of the posterior layer. Posterior component separation, namely Transversus Abdominis Release (TAR) [5] has gained significant popularity due to its outcomes in achieving lengthening of the posterior layer. In W_2-3_ hernias, tension-free posterior midline closure of the posterior layer is often arduous, in the absence of which, the hernia repair has an unfortunate effect of failing and recurring, or resulting in the incarceration of bowel into the retro rectus space. To aid in attaining a tension-free posterior closure, TAR has been employed successfully in open and laparoscopic procedures. It involves incising the posterior lamella of the internal oblique a few centimetres medial to the neuro-vascular bundles and exposing the transversus abdominis muscle. The division of the transversus abdominis muscle along this line allows for lengthening of the posterior layer and can be brought to the midline for closure.

The advantage of TAR is that it allows hernias near bony structures to be addressed—in sub-xiphoid and suprapubic regions.

The main disadvantage of TAR is its technical complexity [8]. When implemented correctly, it offers exceptional results. The technical complexity of TAR may result in profound complications when done outside of large volume centres or without adequate supervised training. Complications though rare, are profoundly morbid when they arise, such as Linea semilunaris injury [15, 16] which leads to denervation of rectus muscle leading to permanent abdominal laxity or iatrogenic spigelian hernia. Normal abdominal wall contour may be difficult to regain. Multiple procedures might be required and additional meshes might have to be placed over the iatrogenic hernia.

Multiple rents may arise during dissection of the transversus abdominis (TA) muscle on the transversalis fascia/peritoneum where the TA muscle belly is thinned out or is tendinous. The peritoneum in large hernias requiring TAR is often thinned out due to the abnormally lax abdominal wall and is susceptible to tearing even on mild traction or manipulation. Rents made need to be closed judiciously [15] and coverage of the mesh over these areas needs to be ensured to provide additional reinforcement to the posterior layer. Such additional operative steps in TAR add significantly to the operative time and financial burden on already stressed healthcare systems.

Injury to diaphragmatic fibres might occur on dissection in the cranial direction, especially in the pre-transversalis or pre-peritoneal plane [17]. This must be avoided at all costs.

Due to the above-mentioned technical difficulties and potential complications in performing TAR, we propose the technique of hernial sac preservation to obviate the need for TAR in select cases of laparoscopic E-TEP repair of midline ventral and incisional hernias. External manipulation is done to bring the sac into the field of vision and grasped using a non-traumatic instrument. The sac is then divided from the anterior abdominal wall using an energy source and is then dissected off the defect. Minor rents may occur during such dissection. The continuity can be restored by intracorporeal suturing. This method has been implemented with good outcomes under the limited follow-up period in the above-mentioned three cases and performing a TAR was prevented.

Though a good technique to have in the toolbox for select cases, in the case of very large hernias, this may be difficult to attempt due to the thinned-out nature of the sac, which will be subject to multiple rents on dissection. This technique requires further evaluation in multicentre studies with a longer duration of follow-up to arrive at a conclusion on criteria of defect size where hernia sac preservation can be attempted in Laparoscopic E-TEP repair of midline ventral and incisional hernias and the performance of TAR can be obviated.

## Data Availability

The original contributions presented in the study are included in the article/Supplementary Material, further inquiries can be directed to the corresponding author.
